# A Ratiometric Fluorescence Amplification Using Copper Nanoclusters with *o*-Phenylenediamine Sensor for Determination of Mercury (II) in Natural Water

**DOI:** 10.3390/s23125429

**Published:** 2023-06-08

**Authors:** Ampika Phoungsiri, Natee Lerdpiriyaskulkij, Pathavuth Monvisade, Ekarat Detsri, Arjnarong Mathaweesansurn

**Affiliations:** 1 Department of Chemistry, School of Science, King Mongkut’s Institute of Technology Ladkrabang, Bangkok 10520, Thailand; 2 Polymer Synthesis and Functional Materials Research Unit, School of Science, King Mongkut’s Institute of Technology Ladkrabang, Bangkok 10520, Thailand; 3 Integrated Applied Chemistry Research Unit, School of Science, King Mongkut’s Institute of Technology Ladkrabang, Bangkok 10520, Thailand; 4 Applied Analytical Chemistry Research Unit, School of Science, King Mongkut’s Institute of Technology Ladkrabang, Bangkok 10520, Thailand

**Keywords:** ratiometric fluorometric method, mercury (II), copper nanoclusters, nanosensor, *o*-phenylenediamine

## Abstract

A simple and rapid method for determining mercury (II) has been developed using L-cysteine-capped copper nanocluster (CuNCs) with *o*-phenylenediamine (OPD) as the sensor. The characteristic fluorescence peak of the synthesized CuNCs was observed at 460 nm. The fluorescence properties of CuNCs were strongly influenced by the addition of mercury (II). Upon addition, CuNCs were oxidized to form Cu^2+^. Then, the OPD were rapidly oxidized by Cu^2+^ to form *o*-phenylenediamine oxide (oxOPD), as evidenced by the strong fluorescence peak at 547 nm, resulting in a decrease in the fluorescence intensity at 460 nm and an increase in the fluorescence intensity at 547 nm. Under optimal conditions, a calibration curve between the fluorescence ratio (I547/I460) and mercury (II) concentration was constructed with a linearity of 0–1000 µg L^−1^. The limit of detection (LOD) and limit of quantification (LOQ) were found at 18.0 µg L^−1^ and 62.0 µg L^−1^, respectively. The recovery percentage was in the range of 96.8–106.4%. The developed method was also compared with the standard ICP-OES method. The results were found to be not significantly different at a 95% confidence level (t_stat_ = 0.365 < t_crit_ = 2.262). This demonstrated that the developed method could be applied for detecting mercury (II) in natural water samples.

## 1. Introduction

Mercury (II) is widely used across many industries, but its global impact as a pollutant demands special attention. Even at low concentrations, mercury is highly toxic to living organisms. Mercury can contaminate natural water sources through human activities. Its biogeochemical cycling allows for accumulation in the food chain, posing serious risks to humans and animals alike. Its toxicity varies based on its form and its ability to accumulate in the environment. The high solubility of mercury (II) makes it the main species found in water [1]. Minamata syndrome is a well-known side effect of mercury (II), which can inflict damage on the central nervous system, mitosis, and DNA [2]. Because of the harmful effects of mercury (II), monitoring its presence in natural water resources is crucial.

Various analytical techniques have been reported for the quantification of mercury (II) in diverse types of specimens, such as chemical vapor generation atomic absorption spectrometry (CVG-AAS) [3,4,5,6], inductively coupled plasma optical emission spectroscopy (ICP-OES) [7,8], atomic fluorescence spectroscopy (AFS) [9], microwave-induced plasma optical emission spectrometry (MIP-OES) [10] and fluorometry [11]. Despite their exceptional sensitivity and selectivity, these methods necessitate expensive and advanced equipment, complicated procedures, and skilled operators.

Recently, nanomaterials have gained significant attention in research as they exhibit distinct physical and chemical characteristics, unlike those of their bulk counterparts [12]. Extensive studies have been conducted on the synthesis of nanomaterials, especially in their application for colorimetric detection of mercury (II) ions. Research has reported various types of nanomaterials, including gold nanoparticles (AuNPs) [13,14], silver nanoparticles (AgNPs) [15], bimetallic nanoparticles [16], and quantum dots (QDs) [17,18,19]. Although these methods provided good sensitivity and selectivity, the synthesis processes comprise multiple steps and are time-consuming, and they also require a final functionalization on their surface. Fluorescent metal nanoclusters, consisting of a few to roughly a hundred atoms, act as bridges between atoms and nanoparticles and exhibit molecular-like properties, such as electron transition, redox property, and intrinsic magnetism [20]. Due to their catalytic properties, high stoke shift, and high quantum efficiency, nanoclusters have been used as chemical sensors [21]. Furthermore, signal amplification strategy has been widely studied in chemical analysis. For example, Z. Sun et al. [22] proposed biosensor based on self-assembled fluorescent AuNPs and duplex-specific nuclease-assisted signal amplification. The probe composed of the modified hairpin DNA with sulfhydryl and fluorescent dye Atto-425 conjugated with the fluorescent AuNPs. The fluorescence of Atto-425 modified on the hairpin DNA was quenched by the AuNPs. The presence of the miR-92a-3p resulted in the removal of Atto-425 from the surface of Au NPs and the recovery of the fluorescence of Atto-425. With the reaction, the fluorescence signal was amplified.

In this work, an efficient fluorescence amplification for the determination of mercury (II) using CuNCs with *o*-phenylenediamine (OPD) as a sensor was reported. The fluorescence of CuNCs was synthesized in an aqueous solution without requiring an additional functionalization process. In the presence of mercury (II), CuNCs were oxidized to form Cu^2+^. The OPD could then be oxidized by Cu^2+^ to generate fluorescent *o*-phenylenediamine oxide (oxOPD), which showed a strong fluorescence peak at 547 nm. This resulted in a dramatic decrease in fluorescence emission at 460 nm, while the fluorescence emission at 547 nm was seen to increase. Based on this detection mechanism, a ratiometric fluorometric method was proposed for determining mercury (II) in natural water.

## 2. Materials and Methods

### 2.1. Chemicals and Reagents

All chemicals used were analytical reagent grade and deionized–distilled water (DI water) was purified using a ZENEER UP 900 (18 MΩ/cm, human corporation, Seoul, Republic of Korea) for use throughout the experiment. Copper nitrate and L-Cysteine were purchased from Sigma-Aldrich, St. Louis, MO, USA. They were employed to synthesize the copper nanocluster. A standard stock mercury (II) solution (100 mg L^−1^) was prepared by dissolving 0.0135 g of mercury chloride (Quality Reagent Chemical, Auckland, New Zealand) in 100.00 mL of DI water. The working standard mercury (II) solutions were prepared by diluting the standard stock solution with DI water. A stock of *o*-phenylenediamine solution (100 mg L^−1^) was prepared by dissolving 0.0100 g of *o*-phenylenediamine (Alfa Aesar, Shanghai, China) in 100.00 mL of DI water. Sodium hydroxide was obtained from Carlo Erba, Milan, Italy. Sodium acetate (Sigma-Aldrich, St. Louis, MO, USA) and acetic acid (Carlo Erba, Italy) were used to prepare a buffer solution. All the glassware used was cleaned via immersion in 10% nitric acid overnight and then rinsed with DI water.

### 2.2. Synthesis of L-Cysteine-Capped Copper Nanocluster

The CuNCs was synthesized according to a previous report [23] with minor modifications. Briefly, 0.1750 g of L-cysteine was dissolved in 5.0 mL of 0.4 M NaOH solution in a beaker through stirring. The 1.0 mL of 1.0 mM copper (II) nitrate was then slowly added dropwise to the solution and stirred for 30 min. After this step, the solution had a pale-yellow color and was heated for 4 h at 55 °C. The resulting dark yellow CuNCs solution was obtained and used without purification. This product could be stored at 4 °C for 3 months with negligible changes to its optical properties (Figure S1).

### 2.3. Sensing Detection of Mercury (II)

Prior to analysis, the synthesized CuNCs colloidal solution was diluted 100-fold with 10 mmol L^−1^ sodium acetate buffer solution (pH 6). The 1.0 mL of different concentration of standard mercury (II) solutions and 75.0 mg L^−1^ OPD were added in fluorometric quartz cuvette. The 1.0 mL of diluted CuNCs solution and the 0.5 mL of copper (II) nitrate solution were then added into the cuvette. After mixing well, the mixture solution reacted at ambient temperatures for 10 min. The fluorescence intensity at 460 and 547 nm was monitored under the excitation wavelength of 375 nm. The calibration plot between the fluorescence intensity ratio (I547/I460) against the standard mercury (II) concentrations was used to quantify mercury (II) in sample solutions.

### 2.4. Detection of Mercury (II) in Real Water Samples

The ratiometric fluorometric method was applied for mercury (II) determination in environmental samples. The water samples were collected from rivers near the Ladkrabang industrial estate in Krung Thep Maha Nakhon (Bangkok, Thailand). The water samples were prepared by filtering them through a 0.22 μm nylon membrane. Then, 25.0 mL of filtered solutions was spiked with the standard mercury (II) solutions at certain concentrations and the volume was adjusted to 50.0 mL with DI water. Instead of the standard mercury (II) solution, 1.0 mL of the sample was added to the solution according to the procedure in Section 2.3.

### 2.5. Validation

The concentration of mercury (II) in the sample was calculated based on the calibration curve, and the obtained results were compared with those measured using inductively coupled plasma optical emission spectrometry (ICP-OES) (Avio 500 Max, PerkinElmer, Waltham, MA, USA). The analytical conditions of ICP-OES were listed as the following parameters: ICP RF power, 1400 watts; plasma argon flow, 15.0 L min^−1^); nebulizer argon, 15.0 (L min^−1^); auxiliary argon, 0.50 (L min^−1^); viewing height, 15 mm; maximum int. time, 40 s; minimum int. time, 4 s; and slit type, normal. The sample preparation for ICP-OES method is described as follows. Firstly, the water samples were filtered through a 0.22 µm membrane. An aliquot of 25.0 mL of filtered samples was spiked with standard mercury (II) solutions at certain concentrations and the volume was adjusted to 50.0 mL with DI water.

## 3. Results and Discussion

### 3.1. Synthesized and Characterization of L-Cysteine-Capped Copper Nanoclusters

**Scheme 1:**
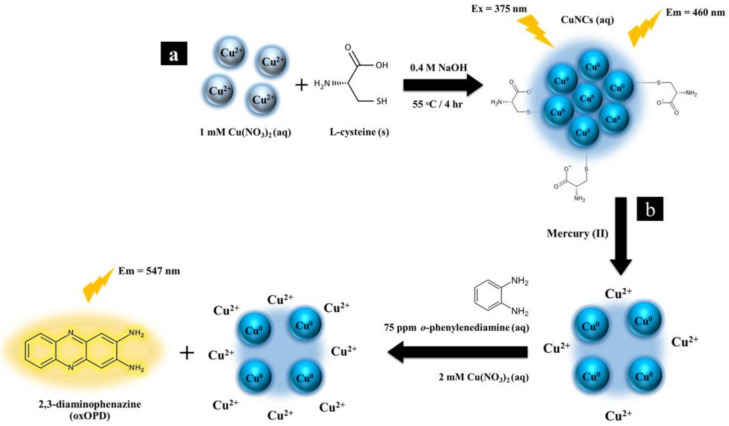
(**a**) Schematic illustration of L-cysteine-capped CuNCs formation and (**b**) illustrated mechanism of L-cysteine-capped CuNCs with *o*-Phenylenediamine (OPD) for the fluorometric detection of mercury (II).

**Figure 1:**
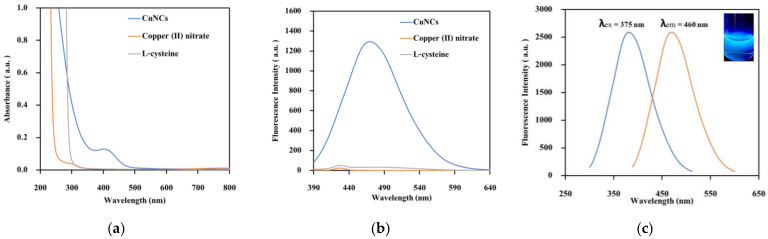
(**a**) UV–vis spectra of L-cysteine-capped CuNCs, L-cysteine and Cu(NO_3_)_2_; (**b**) the fluorescence spectra of L-cysteine-capped CuNCs, L-cysteine and Cu(NO_3_)_2_; and (**c**) the fluorescence excitation and emission spectra of L-cysteine-capped CuNCs.

L-cysteine-capped CuNCs were synthesized according to a very simple chemical reduction method as follows in Scheme 1a. CuNCs were prepared via the reduction in Cu(NO_3_)_2_ with L-cysteine. L-cysteine has been used as both a reducing and stabilizing agent. L-cysteine is a proteinogenic amino acid, which possesses the ability to resonate a lone pair of electrons on sulfur with the thiol (-SH). As is well known, the thiol moiety is a common and popular functional group used for the stabilizing CuNCs in aqueous solutions. The thiol part of L-cysteine binds on the surface of NCs through the Cu–S bond, which provides a cross-linked structure to generate the L-cysteine-modified CuNCs. L-cysteine-capped CuNCs exhibited a weak UV–vis absorption peak around 416 nm (Figure 1a), and both L-cysteine and Cu(NO_3_)_2_ showed no obvious peaks. The maximum excitation and emission spectra of L-cysteine-capped CuNcs were initially recorded as 375 nm and 460 nm (Figure 1b), respectively. In Figure 1c, only the CuNCs exhibited distinct fluorescence properties, indicating that the fluorescence originated from the CuNCs rather than Cu(NO_3_)_2_ or L-cysteine. To explore the surface charges of L-cysteine-capped CuNCs, a zeta potential analyzer was utilized to determine the potential stability in the colloidal CuNCs suspension. The freshly synthesized CuNCs had a mean zeta potential value of −31.6 ± 0.8 mV. This indicated that the CuNCs synthesized with L-cysteine showed a negative zeta potential and were stable at room temperature due to the electrostatic repulsion between the CuNCs, which were enclosed by L-cysteine molecules.

To further elucidate the nanostructures of L-cysteine-capped CuNCs, TEM was employed to directly observe the morphology and particle size distribution. As shown in Figure S2, the CuNCs had a spherical shape with an approximate size of 3.12 ± 0.21 nm.

### 3.2. Sensing Strategy of Copper Nanoclusters with o-Phenylenediamine Sensors for the Determination of Mercury (II)

**Figure 2:**
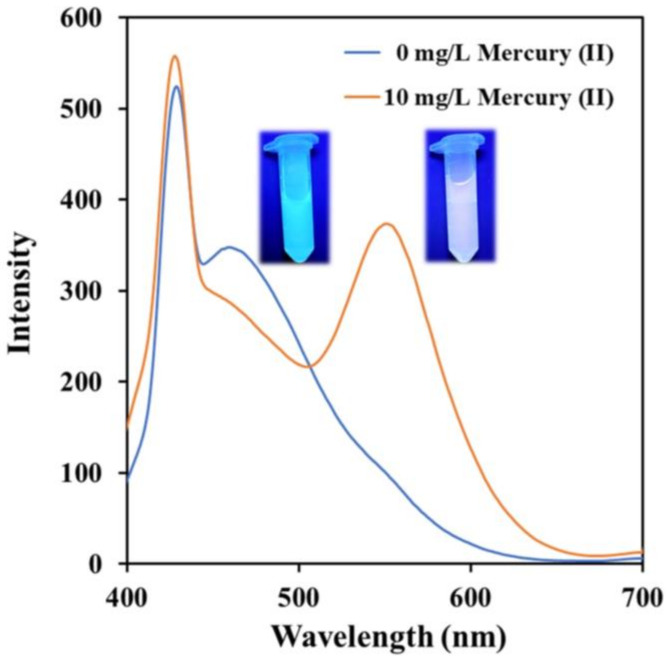
The fluorescence spectra of the sensor in the absence and in the presence of 10 mg L^−1^ mercury (II). The inset photos are the photographs of the sensing solutions under UV light.

Scheme 1b illustrated the sensing mechanism of this method. The surface of CuNCs was modified with L-cysteine, while OPD was used to increase the fluorescence sensitivity of the sensor. The characteristic fluorescence peak of the L-cysteine-capped CuNCs was found to be 460 nm. In the presence of mercury (II), CuNCs were oxidized via mercury (II) to generate Cu^2+^ because the reduction potential of mercury (II) (+0.91 V) is higher than Cu^0^ (+0.34 V). Following the presence of Cu^2+^ from both the oxidized CuNCs and the specific added concentration of the Cu^2+^ source, OPD can be oxidized to form the fluorescent *o*-phenylenediamine oxide (oxOPD), which emits fluorescence at 547 nm. These observations agree with previous reports on the fluorescence enhancement of oxOPD [24]. In addition, when OPD reacted with a mixture of Cu^2+^ and CuNCs, the fluorescence intensity at 547 nm was found to be much greater than when OPD reacted with only Cu^2+^ or CuNCs. Due to the overlap between the fluorescence spectrum of CuNCs and the excitation spectrum of oxOPD, the FRET occurred between CuNCs and oxOPD, resulting in a significant amplification of the fluorescence signal. As a result, the fluorescence intensity at 460 nm decreased while the fluorescence peak at 547 nm increased due to the cooperation of the oxidation reaction and FRET (Figure 2). The color of the colloidal solution under UV light also changed from blue to pale yellow (see inset photos in Figure 2). Based on the detection mechanism, a ratiometric fluorometric method was proposed for the determination of mercury (II). The concentrations of Cu^2+^, CuNCs and OPD were kept constant, and the concentration of mercury (II) was varied in order to examine its effect on the fluorescence intensity. As the mercury (II) concentration increased, the fluorescence intensity at 460 nm decreased and the fluorescence intensity of oxOPD at 547 nm increased. Therefore, the concentration of mercury (II) can be quantified by monitoring the changes in the fluorescence spectra of the mixture solution.

### 3.3. Optimization of the Experimental Conditions

#### 3.3.1. Effect of *o*-Phenylenediamine Concentrations

To optimize the OPD concentration, concentrations ranging from 10 to 100 mg L^−1^ were studied. Table 1 shows the equations for the linear plot of mercury (II) concentration versus intensity ratio at various OPD concentrations. At low concentrations, the OPD reacted with a specific added concentration of Cu^2+^ to produce oxOPD. This resulted in low sensitivity. From Table 1, it can be seen that with the increasing concentration of OPD, the corresponding sensitivity and linearity (R^2^) also increased. The 75.0 mg L^−1^ OPD concentration provided the highest sensitivity and the best linearity. Beyond this concentration, the sensitivity and linearity did not increase further. As a result, the OPD concentration of 75.0 mg L^−1^ was selected as the optimal concentration for further study.

#### 3.3.2. Effect of Copper (II) Nitrate Concentration

**Figure 3:**
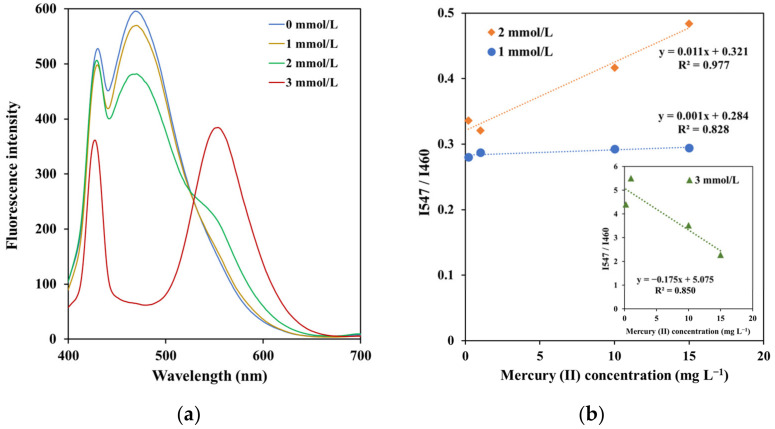
(**a**) The fluorescence spectra of the CuNCs with various copper (II) nitrate concentrations. (**b**) The calibration plots of the intensity ratio at I547/I460 as a function of the mercury (II) concentration when 1.0, 2.0 and 3.0 mmol L^−1^ of copper (II) nitrate concentrations were used.

The effects of the copper (II) nitrate concentrations are presented in Figure 3. According to the literature, the stoichiometric ratio of Cu^2+^ and OPD in the redox reaction is 1:1 [23]. When the copper (II) nitrate was not added to the solution, the oxOPD was not obtained and the characteristic peak of oxOPD at 547 nm was absent. At low concentrations of copper (II) nitrate (1.0 mmol L^−1^), all the Cu^2+^ in the solution was insufficient to react with OPD, causing a decrease in the fluorescence intensity at 460 nm and a slight increase in the oxOPD fluorescence intensity at 547 nm. At high Cu(NO_3_)_2_ concentrations (3.0 mmol L^−1^), OPD was completely oxidized to oxOPD by Cu^2+^. The FRET between oxOPD and CuNCs also occurred, resulting in the appearance of only the fluorescence peak of oxOPD at 547 nm (Figure 3a). Figure 3b showed the calibration plots between the florescence intensity ratio (I547/I460) and Hg (II) concentration for 1.0, 2.0 and 3.0 mmol L^−1^ copper (II) nitrate concentrations. Among these, the 2.0 mmol L^−1^ copper (II) nitrate provided the best linear response (r^2^ = 0.977) and sensitivity. As a result, 2.0 mmol L^−1^ copper (II) nitrate concentration was considered the optimal concentration.

#### 3.3.3. Effect of pH and Reaction Time

**Figure 4:**
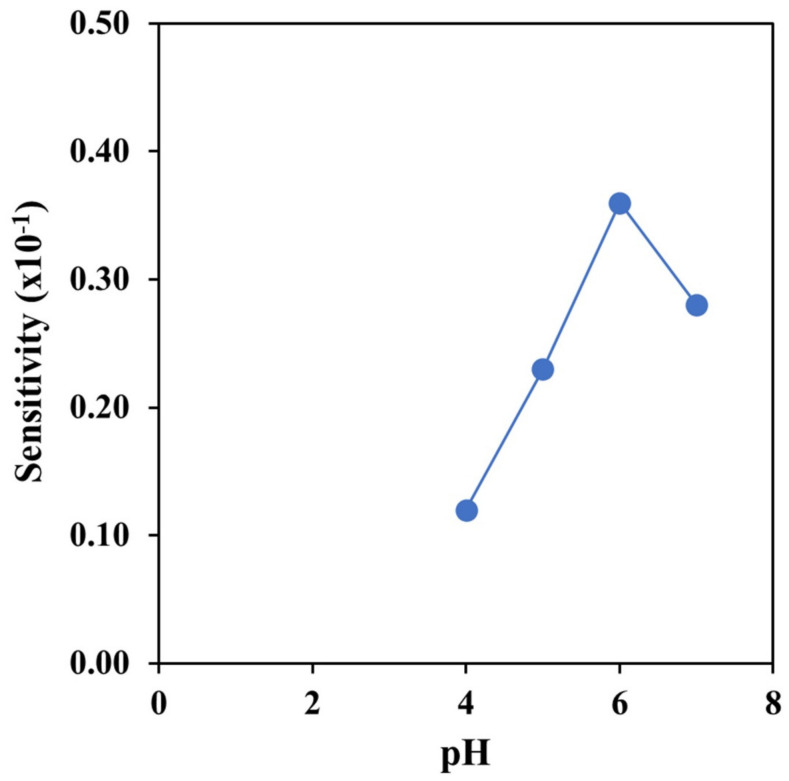
The effect of pH on the sensitivity.

**Figure 5:**
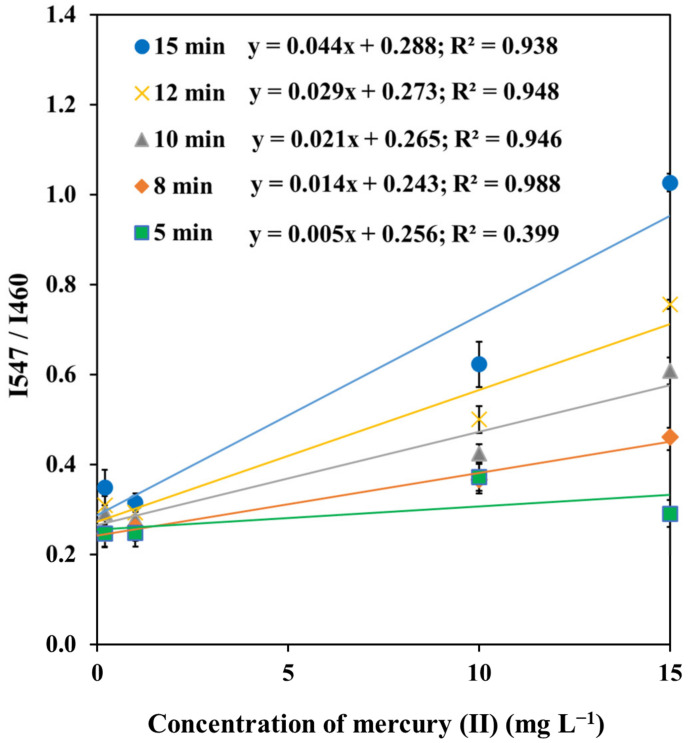
Calibration plots of standard mercury (II) (0–15.0 mg L^−1^) and their corresponded linear correlation coefficients (R^2^) obtained through different the reaction times (5–15 min).

The fluorescence intensity of CuNCs was stable in a wide pH range of 4–9 [25]. The effect of pH on the reaction was studied from 4 to 7 using acetate buffer to adjust the pH of the mixed solution. As shown in Figure 4, the best sensitivity was achieved when the solution pH was 6. The effect of reaction time was also investigated, ranging from 5 to 15 min. The results in Figure 5 showed that the reaction time of 8 min provided the best linear response compared to the other times. After 8 min, the linearity of the calibration curves decreased because of the ongoing oxidation of OPD by the remaining Cu^2+^ in the solution. As a result, 8 min was chosen as the appropriate reaction time.

### 3.4. Method Validation and Fantastic Application

**Figure 6:**
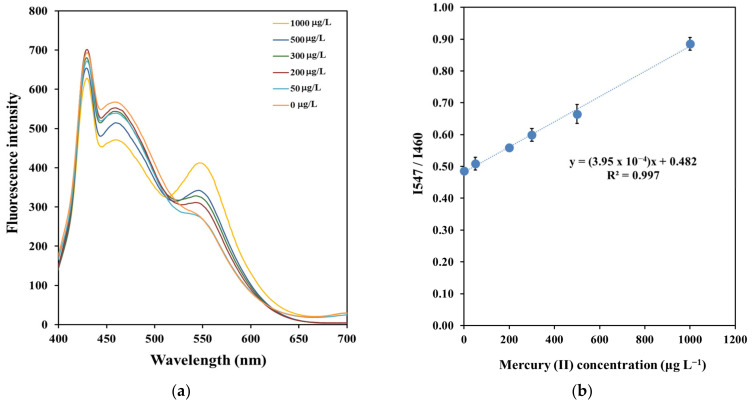
(**a**) The fluorescence spectra of the sensor (containing 75.0 mg L^−1^ of OPD and 2.0 mmol L^−1^ of copper (II) nitrate) at various mercury (II) concentrations. (**b**) The calibration plot of the intensity ratio at I547/I460 as a function of the mercury (II) concentration.

To assess the analytical performance of this method, the validation parameters were investigated under the optimal conditions. For the quantitative analysis of mercury (II), the fluorescence spectrum of the sensing solution was studied by introducing a concentration series of mercury (II) (Figure 6a). The result revealed that the fluorescence intensity decreased at 460 nm and increased at 547 nm with the increase in mercury concentration. The calibration curve was established by plotting the intensity ratio (I547/I460) versus the concentration of mercury (II) (Figure 6b), which demonstrated good linearity in the range of 0–1000 μg L^−1^ (R^2^ = 0.997). The LOD and the LOQ were 18.0 and 62.0 μg L^−1^, respectively. To evaluate the accuracy of the method, the recovery was studied by adding a standard mercury solution to sample solutions to obtain a final added concentration of 50 and 100 μg L^−1^. Table 2 shows that the recovery was in the range of 96.8–106.4%, which is satisfactory. The reproducibility of this method was studied through a series of ten repetitive measurements at concentrations of 10, 300, 500 and 1000 μg L^−1^ mercury. The relative standard deviations (RSD) obtained were 5.6%, 4.2%, 3.8 and 2.2%, respectively, indicating the high accuracy and precision of this developed method.

To verify its effectiveness, the developed method was applied to determine mercury (II) in water samples. The results obtained from the developed method were compared to those obtained from the ICP-OES technique. The mercury (II) content in the samples in Table 2 confirmed that the compared methods are not significantly different under the statistically paired *t*-test (t_stat_ = 0.305, t_crit_ = 2.262) at a 95% confidence level. These results imply that the method was successfully validated and can be applied for the determination of mercury in water samples.

A comparison of the proposed method with the other methods was carried out as shown in Table 3. The analytical features of this method and other methods were compared. The linearity range of this work was better than that of the other methods. The analytical procedure was easier and faster than certain reported methods [5,9,13]. Furthermore, highly expensive instruments were not required for this work.

### 3.5. Selectivity of the Mercury (II) Detection System

**Figure 7:**
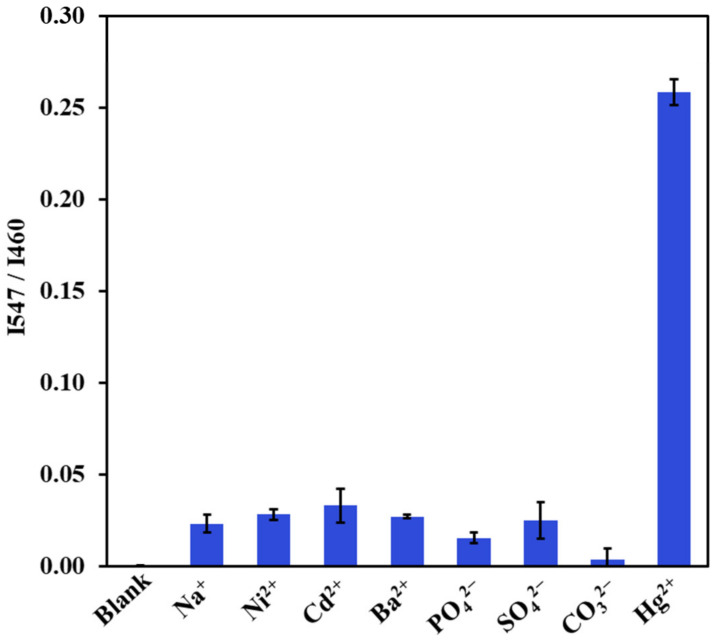
The corresponding I547/I460 ratio of the sensor with the interferences.

In order to investigate the selectivity of this method for mercury (II) detection, certain possibly interfering ions, including Na^+^, Cd^2+^, Ba^2+^, Ni^2+^, CO_3_^2−^, PO_4_^3−^ and SO_4_^2−^ were studied. The concentration of the interference ions used in this investigation was 50 mg L^−1^, which was 100 times higher than that of mercury (II) (0.5 mg L^−1^). Figure 7 shows the intensity ratio versus the interference ions. It is clear that only mercury showed a significant increase in the intensity ratio. These results indicate that these interference ions do not interfere with the assay.

## 4. Conclusions

A signal amplification strategy based on a ratiometric fluorometric method for the determination of mercury (II) using copper nanoclusters with *o*-phenylenediamine as a sensor was proposed here. The fluorescence peak of the L-cysteine-capped CuNCs was located at 460 nm. In the presence of mercury (II), CuNCs were oxidized to Cu^2+^, which then oxidized OPD to the oxOPD form, resulting in a strong fluorescence peak at 547 nm. Due to the overlap between the fluorescence spectrum of CuNCs and the excitation spectrum of oxOPD, the FRET occurred between CuNCs and oxOPD, resulting in a significant amplification of the fluorescence signal. As a result, the fluorescence intensity at 460 nm decreased, while the fluorescence peak at 547 nm increased due to the cooperation of the oxidation reaction and FRET. Based on the detection mechanism, the quantification of mercury (II) was carried out. Under optimal conditions, the determination of mercury (II) was found in a concentration range of 0–1000 µg L^−1^ with excellent linearity. All the results indicated that the proposed method provided high accuracy and high precision and is applicable for determining mercury (II) with good selectivity in real water samples.

## Figures and Tables

**Table 1:** Effect of OPD concentration.

OPD Concentration (mg L^−1^)	Equation	R^2^
10	y = 0.003x + 0.483	0.146
25	y = 0.001x + 0.585	0.141
50	y = 0.004x + 0.832	0.454
75	y = 0.032x + 0.719	0.981
100	y = 0.041x + 0.993	0.865

**Table 2:** Summary on the recovery study of the mercury (II) in water samples.

Sample	Spiked Mercury (II) (μg L^−1^)	Mercury (II) Concentrations (μg L^−1^) (±SD)		Recovery ^a^ (%)
		This Work	ICP-OES	
S-1	50	53.2 ± 0.5	50.7 ± 0.2	106.4
	100	105.7 ± 2.4	98.4 ± 0.8	105.7
S-2	50	51.2 ± 0.3	50.9 ± 0.3	102.4
	100	106.1 ± 4.1	97.8 ± 1.0	106.1
S-3	50	48.4 ± 1.7	52.5 ± 1.1	96.8
	100	97.5 ± 2.9	102.4 ± 0.4	97.5
S-4	50	49.2 ± 2.8	51.3 ± 1.6	98.4
	100	96.9 ± 3.3	103.1 ± 2.1	96.9
S-5	50	51.3 ± 1.4	52.8 ± 1.7	102.6
	100	98.9 ± 5.1	103.7 ± 2.4	98.9

^a^ The recovery percentages in this work.

**Table 3:** Comparison of the proposed method with other analytical methods.

Techniques	Sample	Analysis Time (min)	Linear Range (μg L^−1^)	LOD (μg L^−1^)	%RSD	[Ref]
Sequential injection—CVAAS	Water	0.33	0–60	0.2	1.8	[4]
Photochemical vapor generation—AAS	Naphtha and condensate	30	0–100	0.6	1.2–8.8	[5]
Magnetic on-line solid phase extraction—ICP-OES	Water	4.6	0.2–1000	0.05	1.6	[8]
HPLC-AFS	Tuna fish (CRM)	14	5–200	1.6	50	[9]
Rhodamine B-banded fluorescent chemosensor	Water	0.02	0–6	3.2	2.2–4.9	[11]
AuNPs/UV–vis spectrophotometer	Water	20	2–400	1.0	-	[13]
CuNCs/fluorescence spectrophotometer	Water	8	0–1000	18	2.2–5.8	This work

## Data Availability

Not applicable.

## Supplementary Materials

The following supporting information can be downloaded at: https://www.mdpi.com/article/10.3390/s23125429/s1, Figure S1: The TEM image of copper nanocluster. Figure S2: The TEM image of copper nanocluster.
