# Supplementary material for: A Ratiometric Fluorescence Amplification Using Copper Nanoclusters with o-Phenylenediamine Sensor for Determination of Mercury (II) in Natural Water
Source: Sensors (Basel). 2023 Jun 8;23(12):5429. doi: 10.3390/s23125429 (PMC10302021; doi:10.3390/s23125429)
Supplement: Supplementary file 1 [file sensors-23-05429-s001.zip › sensors-2435087-supplementary.pdf]

Supporting Materials

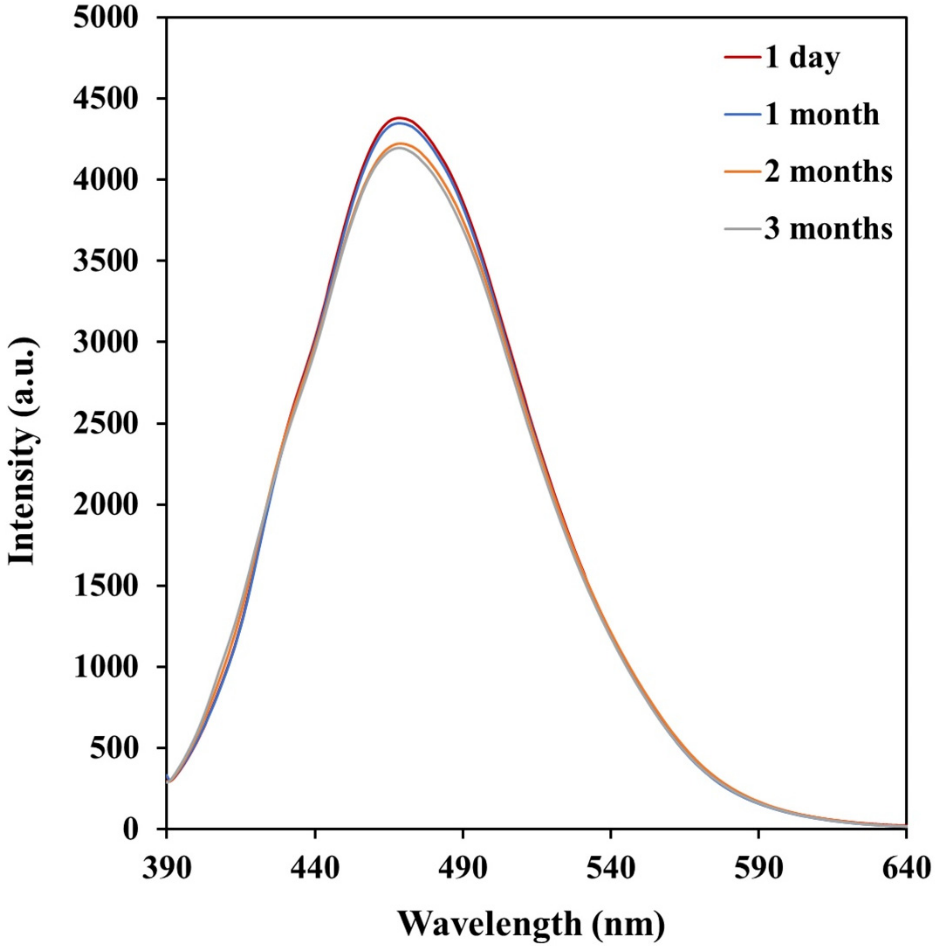

Figure S1. The fluorescence spectra of the CuNCs at different storage times.

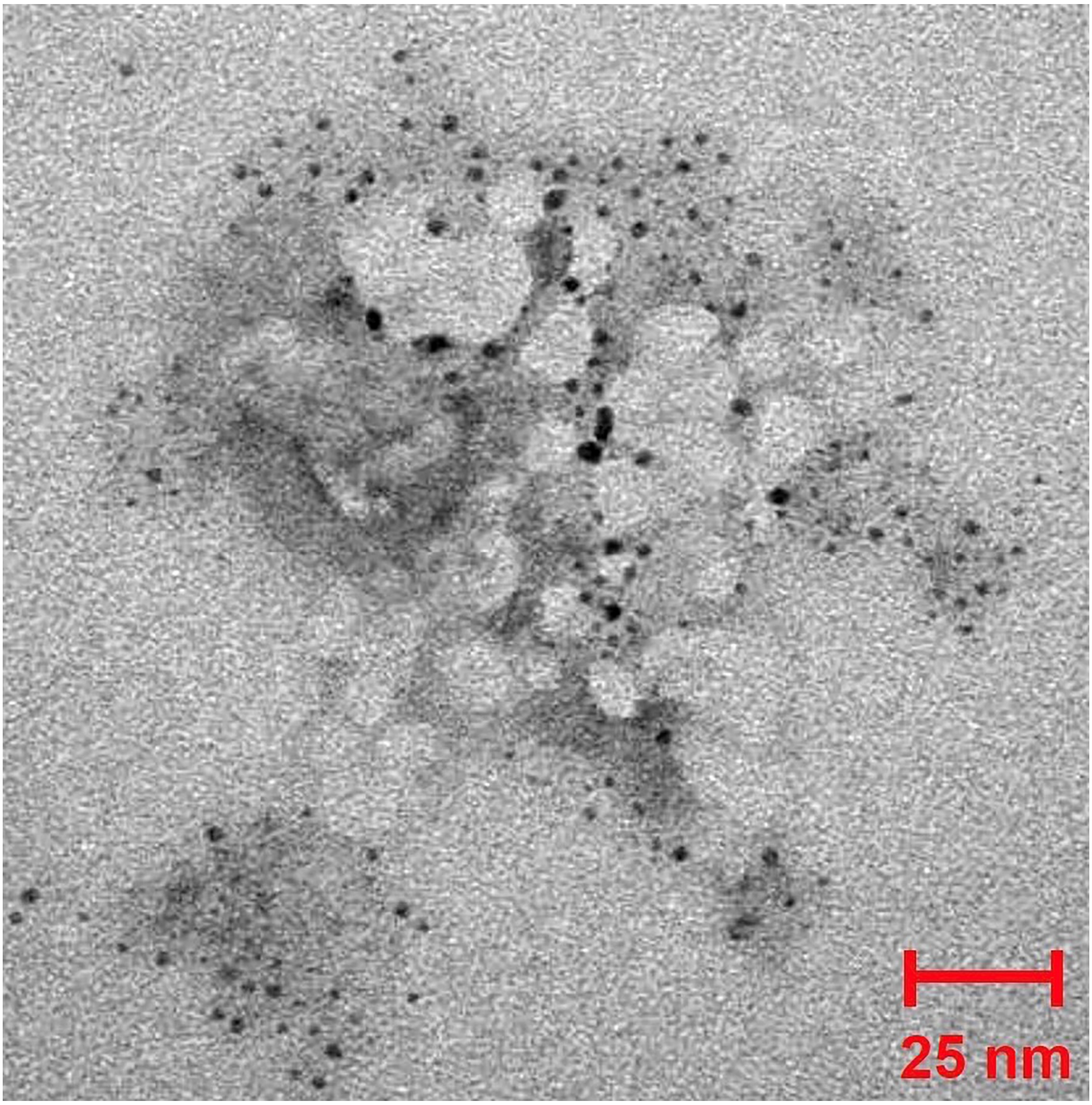

Figure S2 TEM image of the copper nanocluster.
